# High Crystallinity Vertical Few-Layer Graphene Grown Using Template Method Assisted ICPCVD Approach

**DOI:** 10.3390/nano12213746

**Published:** 2022-10-25

**Authors:** Tianzeng Hong, Runze Zhan, Yu Zhang, Shaozhi Deng

**Affiliations:** State Key Laboratory of Optoelectronic Materials and Technologies, Guangdong Province Key Laboratory of Display Material and Technology, School of Electronics and Information Technology, Sun Yat-sen University, Guangzhou 510275, China

**Keywords:** template, crystallinity, vertical few-layer graphene, field emission

## Abstract

Controllable synthesis of high crystallinity, low defects vertical few-layer graphene (VFLG) is significant for its application in electron emission, sensor or energy storage, etc. In this paper, a template method was introduced to grow high crystallinity VFLG (HCVFLG). A copper mask acted as a template which has two effects in the high-density plasma enhanced deposition which are protecting VFLG from ion etching and creating a molecular gas flow to assist efficient growth. Raman and TEM results confirmed the improved crystallinity of VFLG with the assistance of a copper mask. As a field emitter, the HCVFLG has a large field emission current and a low turn-on field. The maximum field emission current of a single HCVFLG sheet reaches 93 μA which is two orders of magnitude higher than VFLG grown without a mask. The maximum current density of HCVFLG film reached 67.15 mA/cm^2^ and is 2.6 times of VFLG grown without a mask. The vacuum breakdown mechanism of HCVFLG was contacted interface damage resulting in VFLG detaching from the substrate. This work provides a practical strategy for high-quality VFLG controllable synthesis and provides a simple method to realize the pattern growth of VFLG.

## 1. Introduction

Vertical few-layer graphene (VFLG) are known to have potential applications in electrical devices, such as sensors, heat management and field emission cold cathode, etc., due to its theoretically excellent thermal, electrical, optical and three-dimension structure [1,2,3,4,5]. Plasma enhanced chemical vapor deposition (PECVD) is the mainstream method to synthesize VFLG including microwave PECVD, direct current PECVD, and induced coupled plasma-enhanced chemical vapor deposition (ICPCVD). ICPCVD has a higher dissociation capacity and higher plasma density (~10^12^/cm^3^) which contains large amounts of radicals. Abundant radicals can improve the nucleation and growth rate of VFLG. In the ICPCVD process, the growth and etching co-exist. Etching is the main reason causing defects on VFLG. VFLG exist large defects, low crystallinity, and weak adhesive problems. Solving the above issues is a key factor to guarantee the realization of the application of VFLG, but the synthesis of high crystallinity VFLG using ICPCVD is challenging and has not been solved. The real performance of VFLG was severely degraded due to defects and weak crystallinity. The measured thermal conductivity of VFLG is only 250 Wm^−1^K^−1^, which is much lower than the few-layer graphene film 1500 Wm^−1^K^−1^ [6,7]. Furthermore, VFLG with defects and weak crystallinity also leads to a sharply decreased electrical conductivity [8,9,10]. Although doping is an efficient way to improve its electrical conductivity [11,12], it is hard to realize heteroatom uniform distribution and accurate doping quantity. In field emission applications, the excellent thermal and electrical conductivity of VFLG is a key factor to realize large current field emissions [13]. Therefore, controllable synthesis of high crystallinity, low defects VFLG is an urgent task.

In the literature on high-quality graphene growth, the state of gas flow is significant for large crystal grain graphene growth. The state of gas flow can be divided into a viscous flow, viscous flow-molecular flow mixed state, and molecular flow according to Knudsen’s number. Under the molecular flow state, intense molecular collision is good for high-quality graphene growth. An efficient way to change gas flow is the limitation of gas flow space [14]. Chen et al. synthesized millimeter-size single-crystal monolayer graphene by adjusting substrate configuration and changing gas flow, such as stacked foil, sandwich structure, or rolled into a cylinder [15]. In the same way, changing the gas flow state is a potential way to synthesize high crystallinity VFLG (HCVFLG). Under high-density plasma circumstances, etching is another key factor influencing the quality of VFLG. High energy ion bombardment can introduce large defects in VFLG during growth. A simple and useful method to avoid etching is using a template as a protector. Yue Qi et al. use copper (Cu) foam as a template reducing ions etching and shielding the electrical field to synthesize flat graphene on a glass substrate by plasma-enhanced chemical vapor deposition [16]. D. I. Tishkevich et al. demonstrated template-assisted method is an efficient way to improve the wettability property of aluminum and can protect deposited Ti/Al_2_O_3_/Ni composite material avoiding corrosion [17,18]. Controllable gas flow and etching are two efficient ways to realize high crystallinity VFLG synthesis.

In this study, a Cu mask was used as a template to synthesize HCVFLG by induced coupled plasma-enhanced chemical vapor deposition (ICPCVD). Gas flow was controlled by adjusting the distance of the Cu mask and substrate. Meanwhile, the Cu mask protects VFLG from etching during growth. The crystallinity of VFLG was characterized by Raman and HRTEM. The underlying growth mechanism of HCVFLG was studied in detail. The electrical and field emission measurement revealed that HCVFLG has excellent field emission performance and great potential in electrical devices.

## 2. Materials and Methods

### 2.1. Preparation of VFLG

The VFLG were prepared using ICPCVD. Cu mask was adopted as a template. Cu foil was used as substrate. Firstly, the Cu substrate was cleaned 10 min using acetone, ethyl alcohol and water, respectively. Secondly, the Cu substrate covered with Cu mesh was placed in the ICPCVD chamber. The chamber was pumped to 2.5 × 10^−2^ Torr by mechanical pump to eliminate air. Meanwhile the substrates were heated to 850 ℃. Thirdly, a gas mixture of H_2_ and CH_4_ with ratio of 10:30 (sccm) was fed into the chamber. The RF power was set as 900 W and the negative bias was adjusted to 150 V to grow VFLG. After 30 mins’ growth, the plasma power and bias voltage were shut down, the gas was turned off. The system temperature was cooled down to room temperature before the samples were delivered.

### 2.2. Characterization

The morphology, structure, composition, and crystallinity of VFLG were characterized by scanning electron microscopy (SEM) (Supra 60, Zeiss), transmission electron microscopy (TEM) (FEI Titan3 G2 60-300), and Raman spectroscopy (In Via Reflex) with 532 nm laser. Field emission characteristics of single VFLG sheet were measured by a nano-probe measurement system in SEM. In this method, a tungsten nano-probe with a radius of 1 μm acted as an anode electrode and the local VFLG under the anode probe were driven to field emission. Then, its field emission characteristics were detected and recorded. Field emission characteristics of VFLG film were measured using anode probe method. The diameter of anode probe was 1 mm. The distance between probe and VFLG film was set as 100 μm. All tests were carried out in DC voltage mode.

## 3. Results and Discussion

**Figure 1:**
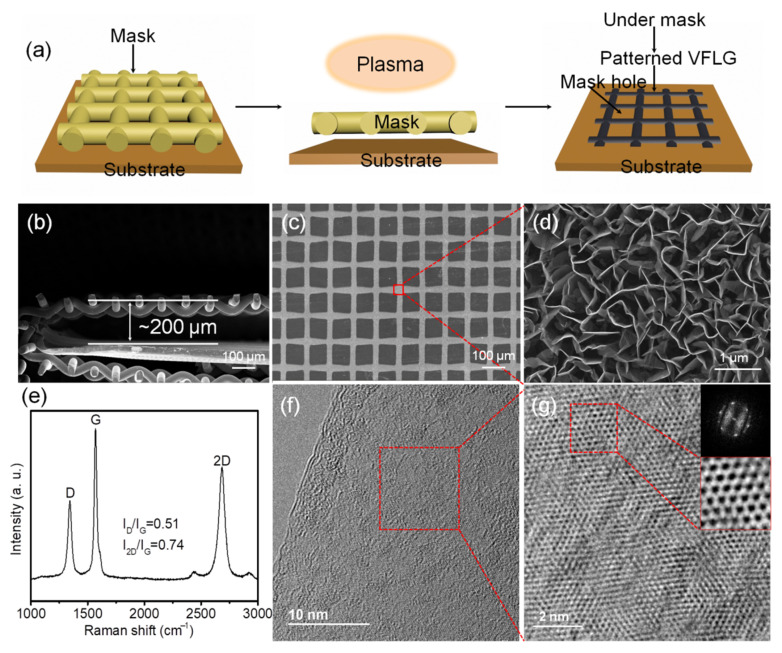
(**a**) Schematic diagrams of HCVFLG preparation. (**b**) SEM image of Cu mask template covered substrate. (**c**) SEM image of HCVFLG and (**d**) high magnification SEM image of the VFLG. Raman spectra of HCVFLG (**e**) and TEM image of HCVFLG (**f**), (**g**) is magnification of the red rectangle in (**f**). Inset of (**g**) is diffraction pattern image and magnification image of the red rectangle area.

Figure 1a shows the schematic diagram of HCVFLG preparation. In the VFLG growth process, etching is the main reason leading to defects and causing low crystallinity of VFLG. The mask covered above the substrate without contact can protect VFLG avoiding the etching of ions bombardment. The distance of mask and substrate was set as a small distance of 200 μm which forms a confined geometry (Figure 1b). The state of gas flow can be changed when through a confined space which is a good way to improve the crystallinity of graphene as proved in the literature [15,19]. The electric field is a necessary factor for VFLG growth. Using a mesh template is a good way to decrease electric field shielding effects and protect VFLG avoiding etching at the same time. As shown in Figure 1c, the VFLG grew under the mask rather than the mesh hole of the Cu mask. The patterned HCVFLG film copied the shape of the Cu mask. The morphology of HCVFLG shows a typical maze structure (Figure 1d). The height, length, and density of HCVFLG are 1 ± 0.14 μm, 0.96 ± 0.16 μm, and 270 ± 34 sheets/100 μm^2^, respectively (more details see Table S1 in Supplementary Materials). The chemical composition of HCVFLG only contains carbon, element Cu comes from the substrate (more details see Figure S1 in Supplementary Materials). Generally, defects and edges of VFLG lead to high-intensity D peaks in Raman spectra. The value of I_D_/I_G_ is usually larger than 1 [20,21,22]. In this experiment, the Raman spectrum of HCVFLG shows a low-intensity of D peak and a thin G peak (Figure 1e). The small I_D_/I_G_ of 0.51 suggests fewer defects with a larger domain zone of graphene. The narrow full width at half maxima (FWHM) of the G peak of 25 means a higher graphitization degree. TEM results show the layer of HCVFLG is about three layers (Figure 1f). No small grains or amorphous structure exists on the HCVFLG sheet. Figure 1g shows the HRTEM image of HCVFLG, the sheet of HCVFLG is flat. A typical honeycomb crystal lattice can be seen clearly in the magnification image (Figure 1g lower inset). The hexagonal diffraction pattern without concentric spheres shows good crystallinity of HCVFLG (Figure 1g upper inset). Different templates were used as masks to synthesize HCVFLG using the above method. Patterned HCVFLG can copy the shape of the template (more details see Figure S2 in Supplementary Materials). This approach provides a new route to synthesize patterned HCVFLG and is meaningful for VFLG application.

**Figure 2:**
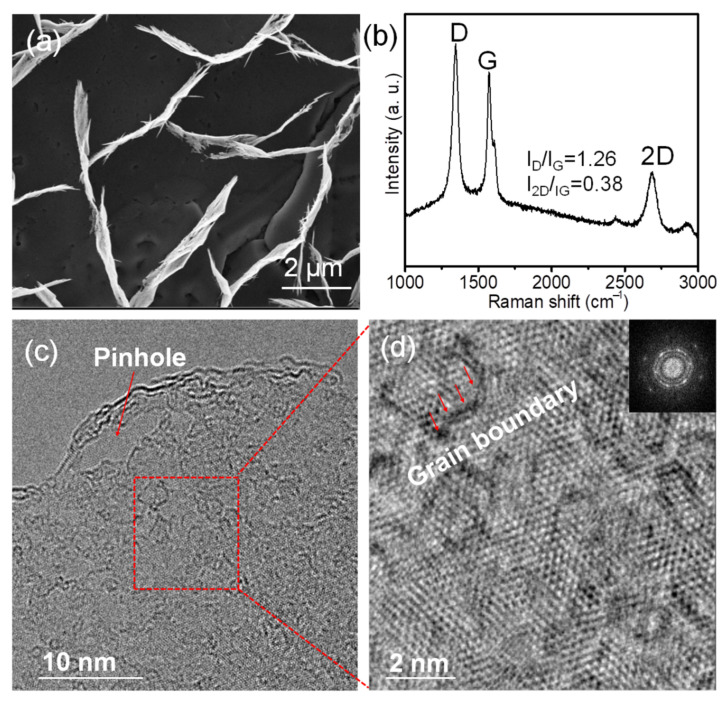
VFLG grown without Cu mask. (**a**) SEM image of the VFLG. (**b**) Raman spectra of the VFLG. (**c**) TEM image of the VFLG. (**d**) is magnification of the red rectangle in (**c**). Inset of (**d**) is its diffraction pattern.

For comparison, VFLG was synthesized without a Cu mask under the same conditions. Figure 2a shows the SEM image of VFLG grown without a mask. The height, length, and density of VFLG are 2.19 ± 0.48 μm, 3.1 ± 0.2 μm, and 27 ± 4 sheets/100 μm^2^, respectively (more details see Table S1 in Supplementary Materials). The density of VFLG grown without a mask is an order of magnitude smaller than HCVFLG. Raman spectra indicate the intensity of the D peak is larger than the G peak. The value of I_D_/I_G_ is 1.26 means a large number of defects exist in the VFLG which is attributed to the etching effect that damages the crystallinity of VFLG and restrains forming large crystal grain (Figure 2b). TEM results show intense ion bombardment introduced a pinhole on the VFLG (Figure 2c red arrow). Small crystalline grains also can be seen clearly in the HRTEM image (Figure 2d). Diffraction patterns with concentric spheres proved the polycrystal structure of the VFLG (Figure 2d inset). The control group results proved that etching can damage the structure of VFLG and restrain crystal grain growth. The template method is an efficient way to avoid etching and realize high crystallinity VFLG.

**Figure 3:**
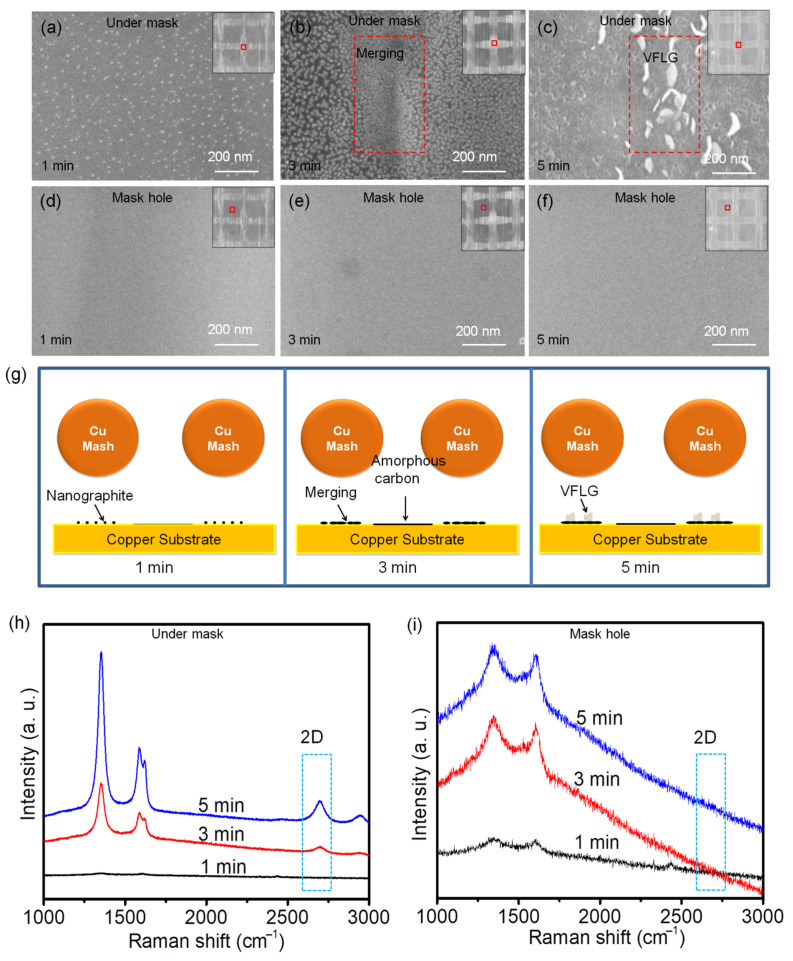
SEM images of VFLG grown with Cu mask in various time; (**a**,**d**) 1 min; (**b**,**e**) 3 min; (**c**,**f**) 5 min. (**g**) Schematic diagrams of VFLG grown 1 min, 3 min, and 5 min; (**h**) Raman spectra of VFLG grown under Cu mask; (**i**) Raman spectra of VFLG grown at Cu mask hole. Inset pictures of (**a**–**f**) show the position of SEM images.

The initial growth process of HCVFLG was studied to understand the growth mechanism. Three HCVFLG samples which grew 1 min, 3 min and 5 min, respectively, were characterized by SEM. As shown in Figure 3a, In 1 min growth, small size and low crystallinity nanographite grew on Cu substrate under the Cu mask which had weak Raman signal without obvious graphene peak (Figure 3h dark line). When the growth time increased to 3 min, the nucleation of nanographite grew up and merged forming a nanographite layer on Cu substrate (Figure 3b red rectangle). The peak of graphene can be seen clearly in Raman spectrum (Figure 3h red line). After 5 min growth, VFLG began to nucleate and grow on the nanographite layer (Figure 3c red rectangle). Raman spectrum shows high-intensity D (1352.4 cm^−1^) peak and D’ (1619.6 cm^−1^) peak corresponding to defects and edges of VFLG (Figure 3h blue line). The schematic diagrams of the VFLG grown 1 min, 3 min, and 5 min is shown in Figure 3g. Carbon radical diffuse and reach on the substrate, then react with each other forming nanographite particles. Nanographite particles grow and merge forming a nanographite layer. VFLG nucleate and grow on the active site of the nanographite layer. In comparison, in the Cu mask hole area, there are no VFLG grown between 1–5 min growth under the same conditions (Figure 3d–f). Raman results show only amorphous carbon deposited on Cu substrate (Figure 3i).

Based on the experimental results above, the mask plays an important role in the synthesis of HCVFLG. One of key function is avoiding the etching of ions. According to Child law, the density of ions current can be described as [23]:

(1) $$J_{0}=\frac{4}{9}\varepsilon_{0}{\left(\frac{2e}{M}\right)}^{\frac{1}{2}}\frac{{V_{0}}^{\frac{3}{2}}}{s^{2}}$$

where *ε*_0_ is permittivity; *e* is elementary charge; *M* is mass of ion; *V*_0_ is bias voltage; *s* is thickness of sheath.

With the bias increasing, the thickness and electric field of the plasma sheath both increase [24]. Ions gain high energy and have intense etching effect after through sheath. According to Child law, ion current increases under high bias. Large amounts of high-energy ions etch Cu substrate directly through the Cu mask hole. Intense etching effect of ions restrains nucleation and growth of VFLG, so no VFLG nucleate and grow on the hole of Cu mask. Under the Cu mask, majority ions are blocked by Cu mask. Nucleation and growth of VFLG are safely going on without ions bombardment. Therefore, fewer defects are introduced which leads to a better crystallinity of VFLG under Cu mask during growth.

Another factor which influences the crystallinity of VFLG is the state of gas flow. Gas flow influences the transport of radicals. According to Knudsen number (*K*_n_), the state of gas flow can be divided into viscous flow (*K*_n_ < 0.1), Knudsen flow (0.1 < *K*_n_ < 10) (viscous flow–molecular flow mixed state) and molecular flow (*K*_n_ > 10). Among the three, molecular flow is best for reaction of radical due to high collision frequency. *K*_n_ can be described as [14]:

(2) $$K_{n}=\frac{\lambda }{L}$$

where λ is mean free path; *L* is diameter of reactor.

The mean free path λ can be described as:

(3) $$\lambda =\frac{k_{B}T}{2^{-\frac{1}{2}}d^{2}p}$$

where *k*_B_ is the Boltzmann constant; *T* is the temperature; *d* is the diameter of gas molecular; *p* is pressure.

**Figure 4:**
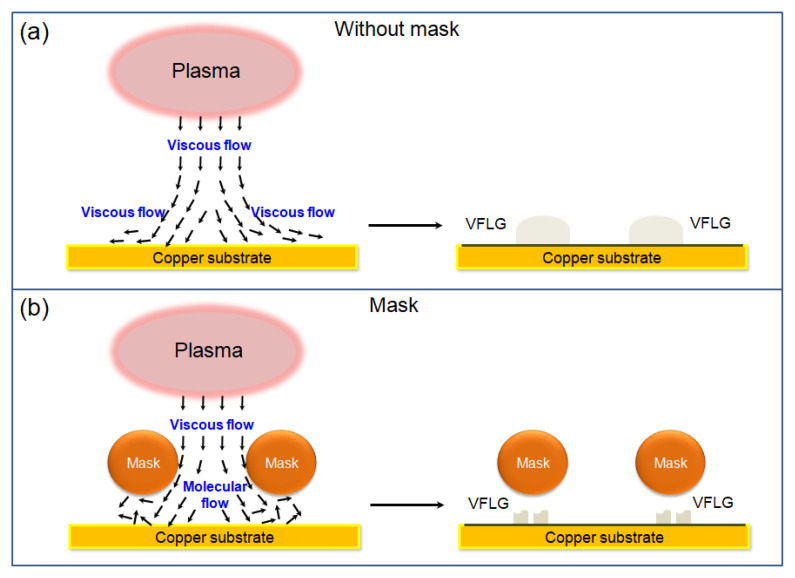
(**a**) Schematic diagrams of VFLG growth without Cu mask which shows a viscous flow on Cu substrate surface; (**b**) schematic diagrams of HCVFLG growth with Cu mask which shows a molecular flow between the Cu mask and Cu substrate.

In our experiment, *T* is 1123 K, *p* is 8 Pa, the diameter of methane molecular is 0.38 nm [25], so λ can be calculated as 6.04 mm. The diameter of the reaction quartz tube is 20 cm. Under above condition, *K*_n_ is 0.03. The state of gas flow is viscous flow due to *K*_n_ < 0.1. In the experimental system, the Cu substrate without Cu mask was exposed to plasma circumstance directly, the state of gas flow is viscous flow. As shown in Figure 4a, ion bombardment can produce more carbon radicals. Large amounts of carbon radicals and a strong electric field of the sheath are good for VFLG growth. However, there is not enough time for the crystallization of carbon radicals due to low reaction rate under viscous flow. Intense ions bombardment restrains the nucleation of VFLG. The low density of VFLG leads to low steric hindrance effect [26,27]. The size of VFLG grows large without a Cu mask, while the Cu substrate under the Cu mask has a small distance of about 200 μm as shown in Figure 4b. Thus, *K*_n_ increases from 0.03 to 30.2. The state of gas flow changes from a viscous flow to a molecular flow on the Cu substrate surface. Radicals are colliding drastically and have high reaction rate under molecular flow state. VFLG has enough time to form high crystallinity grains. Weak etching and enough radicals lead to high nucleation density of VFLG. However, the steric hindrance effect restrains the growth size of VFLG.

Based on the above results, the influence of gas flow on VFLG growth was further studied. The distance between the Cu mask and Cu substrate was increased to 0.5 mm. Thus, *K*_n_ decreased to 10 and the state of gas flow changed from molecular flow to viscous flow–molecular flow mixed state on the Cu substrate surface. The result showed no VFLG nucleated and grew on Cu substrate under the same growth conditions (more details see Figure S3a in Supplementary Materials). Raman results show only amorphous carbon deposited on the Cu substrate (more details see Figure S3b in Supplementary Materials). When the Cu mask and Cu substrate were contacted with each other to form a zero distance, VFLG only grew under the Cu mask’s mesh line which is not well contacted and has a tiny gap (more details see Figure S3c,d in Supplementary Materials). The crystallinity of VFLG became worse due to gas flow obstruction. The above results show the state of gas flow is a key factor for high-quality VFLG growth. Molecular flow is a critical factor for HCVFLG growth.

The electrical conductivity properties of HCVFLG were characterized by a nano-probe measurement system [28]. Resistance of a single HCVFLG sheet and VFLG grown without mask were 4.17 × 10^2^ Ω and 7.5×10^3^ Ω, respectively (more details see Figure S4a in Supplementary Materials). The high crystallinity greatly improves the electrical conductivity of VFLG. The total resistance of HCVFLG and VFLG grown on the Cu substrate were 1 × 10^4^ Ω and 3.4 × 10^4^ Ω (more details see Figure S4b in Supplementary Materials). The resistance of the tungsten probe and Cu substrate was 5 Ω, which means the resistance of the tungsten probe and Cu substrate scarcely influence the total resistance (more details see Figure S4c in Supplementary Materials). The interface resistance between HCVFLG and Cu substrate was the main resistance that determines the total electrical conductivity of the HCVFLG sample.

**Figure 5:**
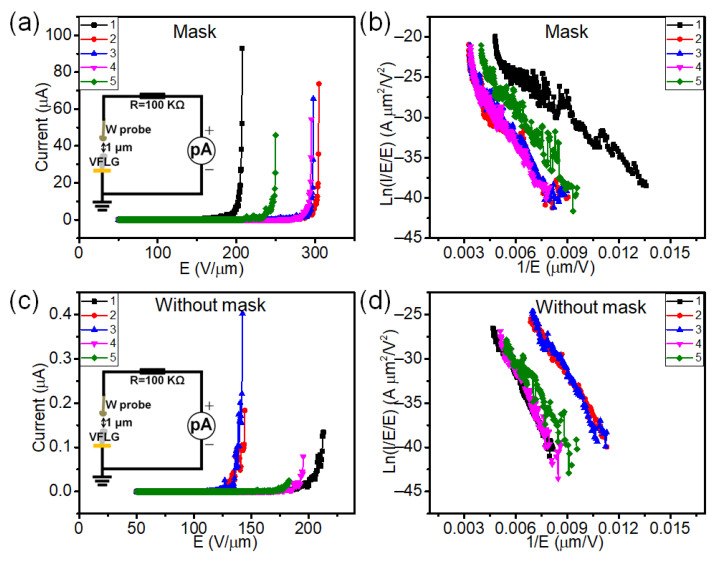
Field emission characteristics of single VFLG sheet. (**a**) Field emission I–V curves of randomly selected five single HCVFLG sheets and corresponding F–N curves (**b**). (**c**) Field emission I–V curves of randomly selected five single VFLG sheets which grow without using Cu mask and corresponding F–N curves (**d**). Inset images of (**a**) and (**c**) are circuits of test. All the tests work in DC voltage mode.

The field emission characteristics of HCVFLG were also measured using the same nano-probe measurement system. The voltage is applied to the anode namely the tungsten probe. The substrate was set as a ground to be a cathode emitter. The distance between VFLG and the probe was 1 μm. The schematic circuit of measurement is shown in Figure 5a,c inset. All the tests work in DC voltage mode. Five randomly selected HCVFLG and VFLG sheets were measured. As shown in Figure 5a, the turn-on field of HCVFLG was 164.4 ± 31 V at a current of 1 nA. The maximum emission current of HCVFLG was 93 μA. In contrast, the turn-on field of VFLG grown without a mask was 146.6 ± 24 V at a current of 1 nA. The maximum emission current of VFLG grown without a mask was only 0.4 μA, which is two orders of magnitude lower than HCVFLG. The corresponding F-N plot is linear in low-field regions and fits with field emission F-N theory (Figure 5b,d). According to F-N theory, the field enhancement factor of HCVFLG and VFLG grown without a mask is 24.41 ± 6.8 and 19.29 ± 2.3, respectively. The low field enhancement factor is due to the small anode–cathode gap. Previously reported studies have proved when the distance between the anode and emitter is very close, the field enhancement factor of emitters decreases with decreasing distance [29]. The results show that the crystallinity of VFLG can contribute to the large current field emission which is essential for many cold electron source applications.

The field emission characteristics of a single HCVFLG sheet are compared with reported works in Table 1. The maximum field emission current of a single HCVFLG sheet is the largest compared with reported works. The high crystallinity of VFLG is good for achieving large current emission characteristics. Moreover, the HCVFLG sheet has the lowest turn-on field compared with the reported graphene sheet which has a similar layer and graphene–anode gap.

**Figure 6:**
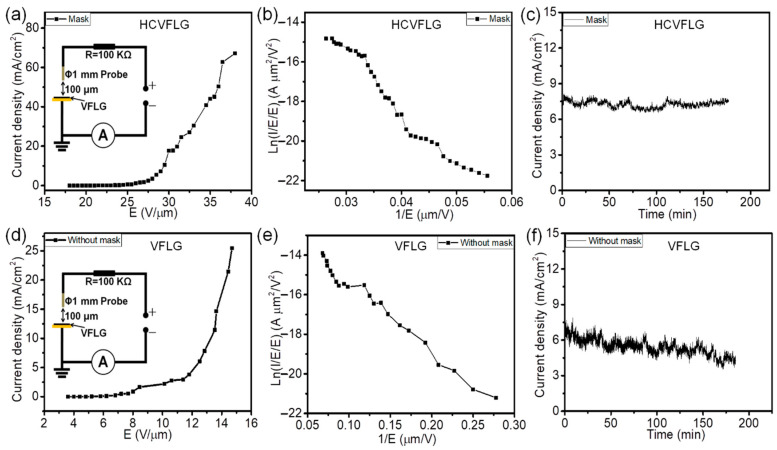
Field emission characteristics of HCVFLG on Cu substrates: (**a**) Field emission I–V curves, (**b**) the corresponding F–N curves, and (**c**) current stability. Field emission characteristics of VFLG grown without mask: (**d**) Field emission I–V curves, (**e**) the corresponding F–N curves, and (**f**) current stability. Inset images of (**a**) and (**d**) are schematic circuits of test.

Field emission characteristics of HCVFLG film (1 × 1 cm) were measured using the anode probe method. The diameter of the anode probe was 1 mm. The distance between the probe and VFLG film was set as 100 μm. All tests were carried out in DC voltage mode. As shown in Figure 6a, the turn-on field of HCVFLG was 18 V/μm at a current density of 0.01 mA/cm^2^. The maximum emission current was 527.13 μA at 38 V/μm; correspondingly, the current density was 67.15 mA/cm^2^. In contrast, the turn-on field of VFLG grown without a mask was 4.8 V/μm at a current density of 0.01 mA/cm^2^ and the maximum emission current was 200 μA at 14.7 V/μm; correspondingly, the current density was 25.48 mA/cm^2^ (Figure 6d). The field enhancement factor of HCVFLG and VFLG grown without a mask was 232 and 1917 (Figure 6b,e), respectively. The field emission stability of the VFLG was performed at 7.5 mA/cm^2^ for 3 h. The current density of HCVFLG scarcely decreased (Figure 6c), the fluctuation is 10.3% (Fluctuation = (I_max_ − I_min_)/(I_max_ + I_min_)). The current density of VFLG grown without a mask decreased from 7.5 mA/cm^2^ to 4.2 mA/cm^2^, decreased 44% (Figure 6f). The high turn-on field and small field enhancement factor of HCVFLG are ascribed to the high density of VFLG. The high density of VFLG causes an intense electric field shielding effect and restrains the field enhancement effect on HCVFLG edges. The density of HCVFLG is larger 10 times compared with VFLG grown without mask (more details see Table S1 in Supplementary Materials). The electric field shielding effect of HCVFLG leads to the field enhancement factor lower about 10 times than VFLG grown without mask. Previous reported studies have proved when the distance is less than the height of emitters, the field enhancement factor of emitters decreases rapidly with decreasing distance, the field emission can be optimized when the distance of emitters is comparable with the high of emitters [35,36].

**Figure 7:**
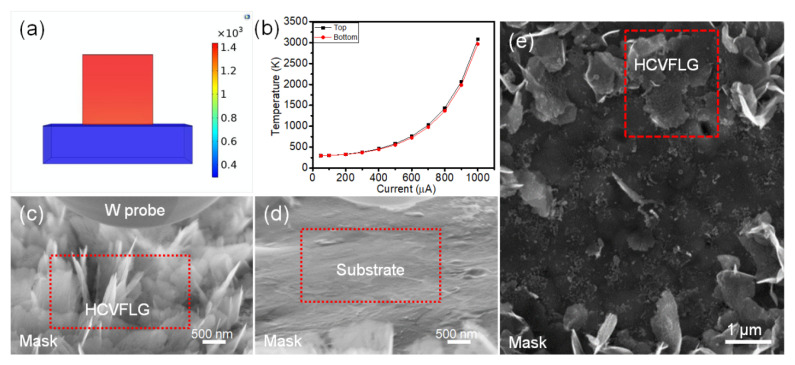
(**a**) Simulation results of the temperature distribution of HCVFLG during field emission. (**b**) Simulation results of the temperature at top and bottom of HCVFLG under different emission current. SEM images of HCVFLG before vacuum breakdown (**c**) and after vacuum breakdown (**d**). (**e**) SEM image of HCVFLG film after field emission test. Red rectangle in (**c**) and (**d**) shows the same area.

A numerical simulation was carried out to understand the vacuum breakdown mechanism of HCVFLG. In this simulation, the substrate was set as Cu and the interface layer was set as the nanographite layer. The height, length, and thickness of VFLG were set as 1 μm, 1 μm, and 2 nm, according to SEM and TEM results. The detailed method and parameters of simulations are described in the Supplementary Materials (more details see note part 1 in Supplementary Materials). As shown in Figure 7a, the temperature distribution of the HCVFLG sheet was similar due to high thermal conductivity and small size. In the simulation, the bottom temperature of the HCVFLG sheet was lower than the Cu melting point (1356 K) at a current of 93 μA (Figure 7b). In the experiment, it was found that the Cu substrate keep its shape after vacuum breakdown of HCVFLG sheets (Figure 7c,d). In HCVFLG film, there are many HCVFLG sheets detached and laid down on Cu substrate after field emission breakdown test (Figure 7e red rectangle). Both the Cu substrate and HCVFLG sheet were scarcely damaged after vacuum breakdown. Therefore, the vacuum breakdown mechanism of HCVFLG film is mainly pointed to the high interface contact resistance which first breaks in high temperature and causes the detachment of HCVFLG from Cu substrate. To realize a high performance HCVFLG field emitter, the improvement of contact interface is the next tough problem in our future work.

## 4. Conclusions

High-quality VFLG has great potential for application in electron emission, sensor, or energy storage due to its theoretically excellent thermal, electrical, and optical properties and three-dimension structure. The crystallinity of VFLG is a key factor to realize its large current field emission. A template method was developed for improving its crystallinity using the ICPCVD method. The template has two effects on the control synthesis of HCVFLG: (1) protecting VFLG avoiding etching during growth; (2) creating the molecular gas flow assisting efficient growth. HCVFLG grown under a Cu mask has low defects and large crystal grains. The electrical conductivity and field emission characteristics of HCVFLG were greatly improved. Compared with the VFLG grown without a mask, the electrical resistance of HCVFLG decreased an order of magnitude and the maximum field emission current increased two orders of magnitude. The vacuum breakdown of HCVFLG is caused by contact interface damage resulting in the detaching of HCVFLG from the substrate. This work reveals a practical way for high-quality VFLG synthesis and also provides a simple method to realize the pattern growth of VFLG.

## Figures and Tables

**Table 1:** Comparison of the field emission characteristics of HCVFLG and the reported graphene sheets.

Graphene Sample	HCVFLG Sheet	Graphene Sheet	Graphene Sheet	N-Doped Graphene Sheet	Multilayer Graphene *	rGO Sheets *
Maximum current (μA)	93	1	1	6.9	60	40
Turn-on field at 1 nA (V/μm)	164.4 ± 31	450	480	275	-	0.1
Breakdown field (V/μm)	207	600	500	525	-	1.36
Layer number	3–5	2	3–10	2–3	14	>10
Sample–anode gap	1 μm	200 nm	200 nm	400 nm	30 mm	22 mm
Ref.	Our work	[30]	[31]	[32]	[33]	[34]

* Large sample–anode gap in mm scale leads to a low turn-on field.

## Data Availability

Data presented in this article are available at request from the corresponding author.

## Supplementary Materials

The following supporting information can be downloaded at: https://www.mdpi.com/article/10.3390/nano12213746/s1, Figure S1: EDS characterization of HCVFLG. The chemical composition of HCVFLG only contains carbon. Element Cu comes from substrate. Figure S2: SEM images of three patterned HCVFLG using different Cu mask. (a) SEM image of HCVFLG growth with circle mesh template. (b) SEM image of HCVFLG growth with line shape template. (c) SEM image of HCVFLG growth with trapezoid mesh template. Figure S3: Influence of gas flow on VFLG growth. The distance of mask and substrate is 0.5 mm: (a) SEM image of VFLG growth 30 min, without VFLG grown on substrate. (b) Raman spectra of VFLG growth 30 min, without the peak of graphene. The mask and substrate contact with each other: (c) SEM image of VFLG growth 30 min, area 1 and 2 without VFLG growth, VFLG grown on area 3. (d) Raman spectra of VFLG growth 30 min. The Raman spectra 1, 2 and 3 correspond to area 1, 2 and 3 in (c). The Raman spectrum of 1 and 2 without see the peak of graphene. The Raman spectrum of 3 has the peak of graphene. Figure S4: Electric conductivity measurement of VFLG: (a) I–V curves of single VFLG sheet cut off from Cu substrates. (b) I–V curve of VFLG on Cu substrate. (c) I–V curve of W probe to Cu substrate. Inset images of (a–c) are schematic circuit of test. Table S1: The morphology parameters of HCVFLG grown with mask and VFLG grown without mask. References [37,38,39,40] are cited in the supplementary materials.
