# Supplementary material for: High Crystallinity Vertical Few-Layer Graphene Grown Using Template Method Assisted ICPCVD Approach
Source: Nanomaterials (Basel). 2022 Oct 25;12(21):3746. doi: 10.3390/nano12213746 (PMC9658688; doi:10.3390/nano12213746)
Supplement: Supplementary file 1 [file nanomaterials-12-03746-s001.zip › nanomaterials-1978346-supplementary.pdf]

Supplementary Materials

High Crystallinity Vertical Few-Layer Graphene Grown Using Template Method Assisted ICPCVD Approach

Tianzeng Hong, Runze Zhan, Yu Zhang * and Shaozhi Deng *

State Key Laboratory of Optoelectronic Materials and Technologies, Guangdong Province Key Laboratory of Display Material and Technology, School of Electronics and Information Technology, Sun Yat-sen University, Guangzhou 510275, China

* Correspondence: stszhyu@mail.sysu.edu.cn (Y.Z.); stsdsz@mail.sysu.edu.cn (S.D.)

Table S1. The morphology parameters of HCVFLG grown with mask and VFLG grown without mask.

Sample	Height (μm)	Layer number	Density (sheets/100μm^{-2})	Length (μm)
HCVFLG	1±0.14	3–5	270±34	0.96±0.16
VFLG	2.19±0.48	3–5	27±4	3.1±0.42

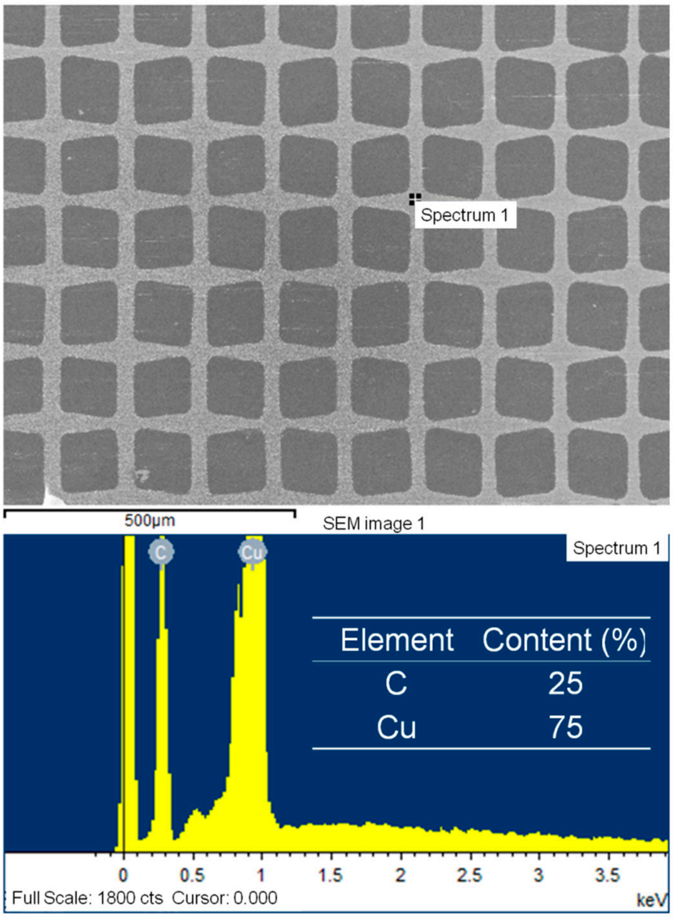

Figure S1. EDS characterization of HCVFLG. The chemical composition of HCVFLG only contains carbon. Element Cu comes from substrate.

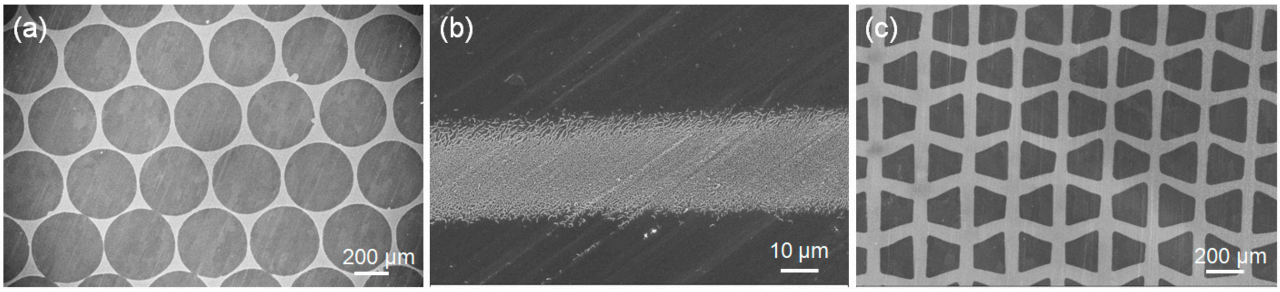

Figure S2. SEM images of three patterned HCVFLG using different Cu mask. (**a**) SEM image of HCVFLG growth with circle mesh template. (**b**) SEM image of HCVFLG growth with line shape template. (**c**) SEM image of HCVFLG growth with trapezoid mesh template.

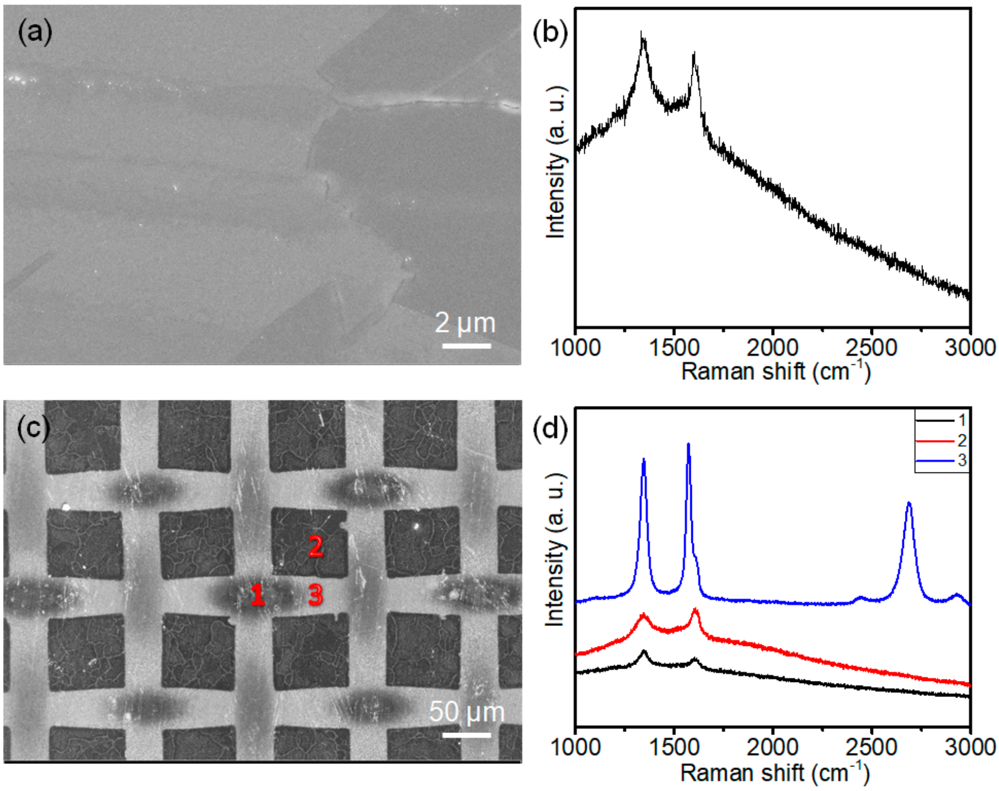

Figure S3. Influence of gas flow on VFLG growth. The distance of mask and substrate is 0.5 mm: (**a**) SEM image of VFLG growth 30 min, without VFLG grown on substrate. (**b**) Raman spectra of VFLG growth 30 min, without the peak of graphene. The mask and substrate contact with each other: (**c**) SEM image of VFLG growth 30 min, area 1 and 2 without VFLG growth, VFLG grown on area 3. (**d**) Raman spectra of VFLG growth 30 min. The Raman spectra 1, 2 and 3 correspond to area 1, 2 and 3 in (**c**). The Raman spectrum of 1 and 2 without see the peak of graphene. The Raman spectrum of 3 has the peak of graphene.

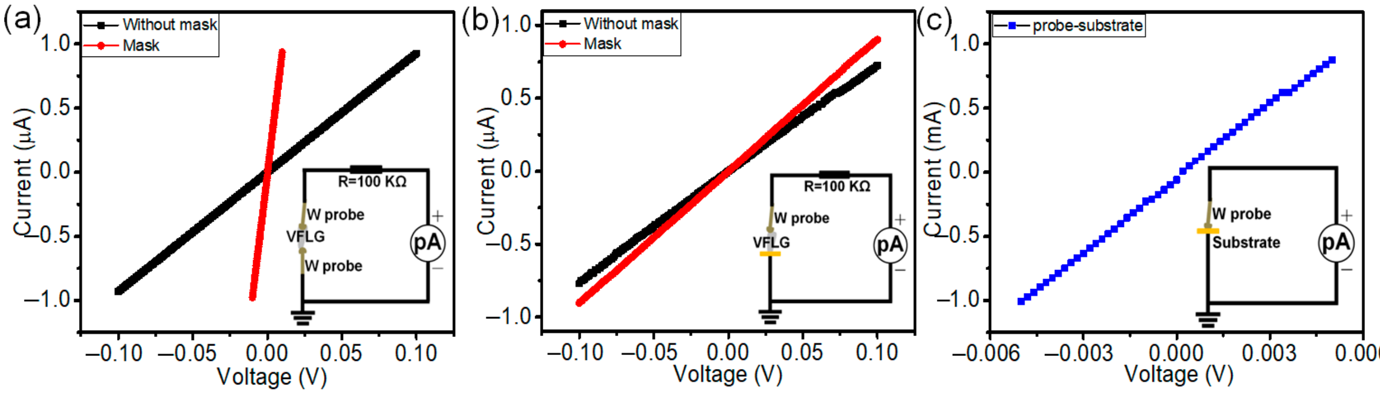

Figure S4. Electric conductivity measurement of VFLG: (**a**) I–V curves of single VFLG sheet cut off from Cu substrates. (**b**) I–V curve of VFLG on Cu substrate. (**c**) I–V curve of W probe to Cu substrate. Inset images of (**a**–**c**) are schematic circuit of test.

1. Temperature distribution simulation of VFLG during field emission

The simulations were performed by COMSOL Multiphysics software. The heat transfer in solids and electric currents modules were used for the temperature distribution of VFLG during field emission. The substrate was set as Cu and the interface layer was set as nanographite layer. The height, length and thickness of VFLG were set as 1 μm, 1 μm and 2 nm respectively. The conductivity of VFLG is calculated by those two equations below:

$$\sigma = 1/\rho$$

$$\Omega = V/I = \varrho l / S$$

where σ is the electrical conductivity, ϱ is the electrical resistivity, Ω is the electrical resistance, V is the voltage, I is the current, S is the cross-section area, *l* is length. The resistance is obtained from I–V curve of high crystallinity VFLG (Figure S3 (b)). The cross-section area S and length *l* are obtained from SEM and TEM image. Using this method, the conductivity of HCVFLG was calculated to be 1100000 S/m. The thermal conductivity of HCVFLG, nanographite layer and Cu substrate were 1100 W/k•m, 10 W/k•m and 400 W/k•m respectively [1–3]. The initial temperature of Cu substrate is set as room temperature 293 K. The heat balance equation can be described as [4]:

$$J \bullet E - \nabla \bullet (k \nabla T) - \varepsilon \sigma S (T_{amb}^4 - T^4) = 0$$

Where *J* is electric current density, *E* is electric field, *k* is heat conduction coefficient, *ε* is surface emissivity, *σ* is Stefan–Boltzmann constant, *S* is surface area, T_{amb} is ambient temperature. Three terms are corresponding heat generation, heat conduction and heat radiation of VFLG.

Reference

1. Murali, R.; Yang, Y.; Brenner, K.; Beck, T.; Meindl, J.D. Breakdown Current Density of Graphene Nanoribbons. *Appl. Phys. Lett.* **2009**, *94*, 243114.
2. Alexander, A.B. Thermal properties of graphene and nanostructured carbon materials. *Nat. Mater.* **2011**, *10*, 569–581.
3. Shul'zhenko, A.A.; Sokolov, A.N.; Jaworska, L.; Gargin, V.G.; Kuz'menko, E.F. Thermal Conductivity of Copper with the Addition of n-Layer Graphene. *J. Superhard Mater.* **2019**, *41*, 283–285.
4. Guo, C.; Xu, N.S.; Zhang, Y.; Ke, Y.L.; Chen, J.; She, J.C.; Deng, S.Z. One-step Growth of Graphene-Carbon Nanotube Trees on 4'' Substrate and Characteristics of Single Individual Tree. *Carbon* **2017**, *125*, 189–198.
